# Continuity of care among diabetic patients in Accra, Ghana

**DOI:** 10.3389/fpubh.2023.1141080

**Published:** 2023-05-09

**Authors:** Veronica Awumee, Samuel Kennedy Kangtabe Dery

**Affiliations:** ^1^Department of Population, Family and Reproductive Health, School of Public Health, University of Ghana, Legon, Ghana; ^2^Department of Biostatistics, School of Public Health, University of Ghana, Legon, Ghana

**Keywords:** diabetes mellitus, continuity of care, longitudinal continuity, relational continuity, Ghana, Accra

## Abstract

**Introduction:**

Diabetes mellitus is a fast-rising non-contagious disease of global importance that remains a leading cause of indisposition and death. Evidence shows that effective management of diabetes has a close link with continuity of care which is known to be the integral pillar of quality care. This study, therefore, sought to determine the extent of continuity of care between diabetic patients and their care providers as well as factors associated with relational continuity of care.

**Methodology:**

This cross-sectional, facility-based study was conducted among diabetics in Accra, Ghana. We sampled 401 diabetic patients from three diabetic clinics in the region using a stratified and systematic random sampling technique. Data were collected using a structured questionnaire containing information on socio-demographic characteristics, the four dimensions of continuity of care, and patients' satisfaction. A 5-point Likert scale was used to measure patient's perception of relational, flexible, and team continuity, while most frequent provider continuity was used to measure longitudinal continuity of care. Scores were added for each person and divided by the highest possible score for each domain to estimate the continuity of care index. Data were collected and exported to Stata 15 for analysis.

**Results:**

The results show that team continuity was the highest (0.9), followed by relational and flexibility continuity of care (0.8), and longitudinal continuity of care was the least (0.5). Majority of patients experienced high team (97.3%), relational (68.1%), and flexible (65.3%) continuity of care. Most patients (98.3%) were satisfied with the diabetes care they received from healthcare providers. Female subjects had higher odds of experiencing relational continuity of care as compared to male subjects. Furthermore, participants with higher educational levels were five times more likely to experience relational continuity of care than those with lower educational background.

**Conclusion:**

The study demonstrated that the majority of diabetics had team continuity of care being the highest experienced among the four domains, followed by flexible and longitudinal being the least experienced. Notably, team and flexible continuity of care had a positive association with relational continuity of care. Higher educational level and being female were associated with relational continuity of care. There is therefore the need for policy action on the adoption of multidisciplinary team-based care.

## Introduction

Diabetes mellitus (DM) remains a major public health burden in Ghana. The occurrence of diabetes in Ghana is increasing rapidly, coupled with high complications, admission, and fatalities. For instance, the prevalence of type 2 diabetes mellitus (T2DM) among urban men and women stands at 10.3 and 9.2%, respectively (1). This is coupled with the prevalence of high chronic complications including glycemic complications (79%), cerebrovascular conditions (10.5%), renal impairment/ nephropathies (18.3%), cardiovascular condition (21%), peripheral neuropathy (60.4%), diabetic foot disease (4.9%), eye disease (58.6%), and erectile dysfunction (31.0%). Consequently, diabetes admission rates has seen increase of more than 6-folds and inpatient fatality from 7.6 per 1,000 deaths in 1983 to 30 per 1,000 deaths in 2012, with an average 28-day mortality rate of 18.5% (2, 3). Moreover, the prevalence of hypertension either as a comorbid or complication in diabetics remains astronomically high, 97.2% (2).

The term “continuity of care” evolved in the 1960s and the concept is still evolving. In the healthcare literature, the term has been used to describe several relationships between patients and providers in the delivery of care services. These definitions have evolved and overlap with concepts such as coordination, integration, and patient-centered care. Even though there is no universally accepted definition of continuity of care, there is general acceptance that, it is a multi-dimensional concept and as a result, several authors have proposed several terms to describe the various dimensions involved (4–6). It can best be described as a “hierarchical concept ranging from the basic availability of information about the patient's past to a complex interpersonal relationship between physician and patient characterized by trust and a sense of responsibility” (6). Table 1 summarizes the key dimensions that have been proposed.

**Table 1:** Dimensions of continuity of care.

**Dimension**	**Description**
Longitudinal/chronological continuity	Care from a regular site of care (5–11)
Relational/interpersonal continuity	Ongoing relationship between a patient and the healthcare providers (6–9)
Information continuity	Availability of and shared information between healthcare professionals (5–12)
Team continuity	Good communication across a team of professionals or services (13)
Management continuity	A consistent approach to the management of a patient from all those involved (4)
Geographic continuity	Care that is given or received in person on one site (office, home, hospital, etc.) (6)
Site continuity/clinician continuity	Care from multiple but related physicians such as those practicing as a group (9, 12)
Referral continuity	Care linked by a referral (9)
Flexible continuity	Services that are flexible and adjusted to the needs of the individual over time (13).
Cross-boundary continuity	Care that follows the patient across settings (e.g., from primary care to hospital or vice versa) (13)
Structural continuity	”Site of medical encounter and the way in which the delivery of services is organized” Nassif et al. (14)
Process continuity	“The coordinated delivery of care over a period of time or throughout an illness episode“ Nassif et al. (14)

Diabetes mellitus is a long-term condition, and its management requires regular interaction between the patient and the care provider. There is evidence that increasing patients–clinicians interaction results in better treatment outcomes (6, 15), and that increased continuity of care leads to a lower mortality rate (16). Relational continuity of care is characterized by an ongoing personal relationship between the patient and care provider guided by personal trust and a sense of responsibility (6). This interpersonal relationship has been shown to improve patients' adherence to treatment and cooperation with care providers (17). The development of this interpersonal relationship requires frequent and repeated visits of patients to their usual care providers (longitudinal continuity), responsive care in the face of changing needs of the patient (flexible continuity), and well-coordinated care (team continuity) (18–20). Similar evidence by Gulliford et al. (21) shows that to achieve optimal treatment objectives for diabetes, there must be an existing bond between diabetes patients and their care givers for the entire treatment process. However, there is a lack of comprehensive evidence to establish the extent of the four dimensions of continuity of care experienced and the factors influencing relation continuity of care between diabetics and their care providers in Ghana. A clear understanding of the effect of longitudinal continuity of care on relational continuity (and vis visa) among diabetes mellitus patients and their health providers in Ghana will help inform and reshape policy regarding treatment and management of the condition. This study, therefore, sought to determine the extent of continuity (relational, longitudinal, flexible, and team) of care and factors associated with relational continuity of care among diabetics and their care providers in three health facilities in the Greater Accra Region of Ghana.

## Methods

### Study design

The study employed a cross-sectional, health facility-based design using a quantitative data collection approach to collect data. This study involved a survey with a sample size of 401 diabetic patients from three health facilities (La-General hospital in the La-Dadekotopon Municipality, Pentecost hospital in the La-Nkwantanang Municipality and the Cocoa clinic in the Accra Metropolis) in the Greater Accra region of Ghana in 2019. Diabetic patients attending the outpatient department (OPD) of the selected facilities and on medication for at least 12 months preceding the study were included.

#### Measurement of continuity of care

In this study, continuity of care was measured using composite indices which were generated from questions that measured each of the four dimensions of continuity. A 5-point Likert scale was used to measure patients' perception of relational, flexible, and team continuity of care. Four sets of variables (“there exist a strong interpersonal relationship between me and my doctor,” “my doctor knows my familial circumstances very well,” “my doctor is concerned about me,” and “my doctor knows my daily activities very well”) were used to measure relational COC. Three variables were used to measure flexible COC (“it is easy to communicate with my health provider about my diabetes,” “I must wait for a long period of time before I speak with a doctor or nurse at the hospital for my diabetes care,” and “it does not take long to obtain an advice urgently from a doctor or nurse”). For team COC, seven sets of variables (“in general, my diabetes care is well-coordinated,” “these health providers transfer information very well to each other,” “these health providers work together very well,” “they share an agreed plan of treatment for my diabetes care,” “the care of these health providers is very well-connected,” “the health providers know very well from each other what they do,” and “I feel the healthcare providers communicate well with each other whenever I visit the hospital”) were used. To estimate the scores for flexible, relational, and team continuity, items under these three dimensions were rated from 1 to 5 points (from strongly disagree to strongly agree). Scores of the responses were added for each person and divided by the highest possible score for each dimension to estimate the continuity index (22). These continuity indices were also categorized into low (<0.75) and high (≥0.75) for each dimension (23).

Similarly, longitudinal continuity of care was measured using the most frequent provider continuity of care (MFPC) index. This is a measure of the extent of concentration or spread of the patient's visits among different physicians. This was computed by determining the proportion of visits to the regular provider out of all visits to the healthcare physician for the past 12 months.


$$\begin{array}{l} MFPC=\frac{\max\left(n1,\;n2,\;n3\ldots \ldots .nk\right)-1}{N-\;1} \end{array}$$

where max (n1, n2, n3,…., nk) is the number of visits to the most frequently visited provider and N is the total number of visits (6).

The values for this index range from 0 (no visit to the regular provider) to 1 (all visits made to the regular provider). The values were transformed into categorical variables and further categorized into two sub-sections based on the distribution of the scores low (<0.75) and high (≥0.75) (19).

#### Data collection and analysis

A structured questionnaire was used to collect data on socio-demographics, patients' experience with longitudinal, flexible, relational, team continuity of care, and patients' satisfaction with diabetes care. The completed questionnaires were captured and cleaned using Microsoft Excel. Descriptive statistics such as frequency distribution, proportions, and charts were used for the categorical variables, while mean scores and their respective standard deviations were computed for continuous variables. Multiple logistic regression analysis was used to measure the strength of the association between relational continuity and other independent variables. This was done by first running a bivariate analysis between all the domains of continuity (longitudinal, relational, flexible, and team continuity and patient satisfaction) and all other independent variables. Independent variables with a *p*-value of ≤ 0.05 in bivariate analysis were fitted in the final multiple logistic regression model to assess the strength of association using the adjusted odds ratio (AOR) with a 95% confidence interval (CI).

## Results

### Demographic characteristics

Four hundred and one (401) participants took part in the study with the mean (±SD) age of all participants being 61.6 ± 11. The majority of patients were female subjects (73.1%), one-third had some formal education, and 20.7% had no formal education. Regarding employment, more than half (71.6%) were employed in the informal sector, while 5.5% were employed in the formal sector and 20% were retired. Most participants were currently married (77.6%), and the majority were Christians (76.8%). In addition, 66.1% indicated they had a family history of diabetes, while almost all (99.5%) stated they had health insurance as shown in Table 2.

**Table 2:** Demographic characteristics of respondents.

**Variable**	**Frequency**	**Percentage**
**Age (years)**		
Mean (SD)	**61.6 (11.1)**	
<40	10	2.5
40–49	46	11.5
50–59	116	28.9
60–69	133	33.2
70+	96	23.9
**Sex**		
Male	108	26.9
Female	293	73.1
**Level of education**		
None	83	20.7
Primary	40	10.0
JSS/JHS/middle school	144	35.9
SSS/SHS/O and A “level”	60	15.0
Technical/vocational	54	13.4
Tertiary	20	5.0
**Occupation**		
Unemployed	13	3.2
Formal sector worker	22	5.5
Informal sector worker	287	71.6
Retired	79	19.7
**Marital status**		
Single	7	1.7
Currently married	311	77.6
Currently not married	83	20.7
**Religion**
Christianity	308	76.8
Islam	93	23.2
**Family history of diabetes**		
Yes	265	66.1
No	136	33.9
**Health insurance**		
Yes	399	99.5
No	2	0.5
**Type of insurance**		
NHIS	388	97.2
Private insurance	11	2.8

Mean (SD): mean (standard deviation).

### The extent of continuity of care

**Figure 1:**
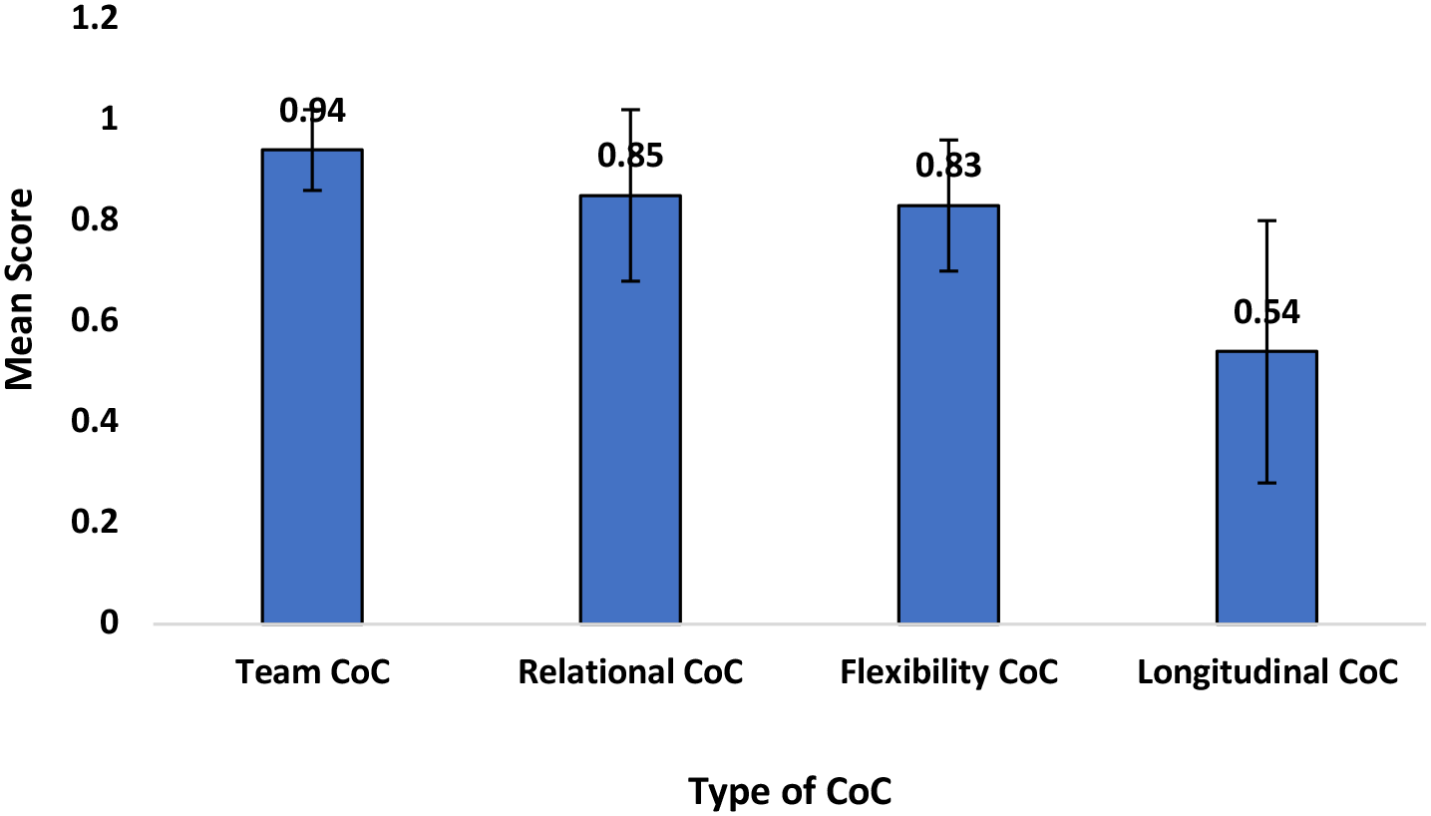
Extent of continuity of care.

The extent of COC is summarized in Figure 1. The highest extent of continuity of care was team continuity of care, while longitudinal continuity of care was the least experienced by patients (0.5).

**Figure 2:**
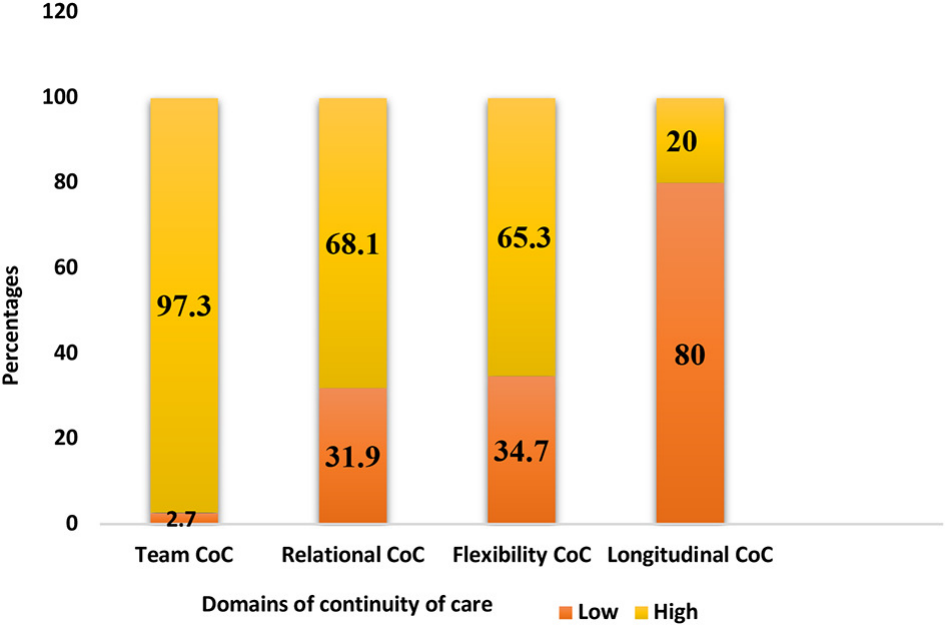
Proportion of respondents by continuity of care dimension.

Figure 2 below summarized the proportion of respondents and the extent of continuity in each of the four dimensions of COC. The majority (97.3%) of the respondents had high team continuity, while 68.1% had high relational continuity. Furthermore, 65.3% of respondents had high flexible with only 20% with high longitudinal continuity.

### Factors associated with relational continuity of care

Binary logistic regression was performed to determine factors associated with relational continuity of care. Patients' adherence to management, and flexible and longitudinal continuity of care were factors associated with high relational continuity of care. The logistic regression model showed that patients with good adherence to the management of diabetes were two times more likely to experience high relational continuity of care (AOR = 2.10; 95% CI: 1.02–4.34; *p* = 0.045) compared to those with poor adherence. The model also revealed that diabetics with high flexible continuity of care were also more likely to have high relational continuity of care (AOR = 5.10; 95% CI: 3.10–8.38; *p* < 0.001) compared to those with low flexible continuity. However, patients with high longitudinal continuity of care were 71% less likely to experience high relational continuity of care (AOR = 0.29; 95% CI: 0.16–0.52; *p* < 0.001). Though not statistically significant, patients who were satisfied with diabetes care were 21% more likely to have high relational continuity of care (AOR = 1.21; 95% CI: 0.06–26.18; *p* = 0.901) as shown in Table 3.

**Table 3:** Factors associated with relational continuity of care.

**Variable**	**COR (95% CI) *p*-value**	**AOR (95% CI) *p*-value**
**Age (years)**		
<40	Ref	
40–49	0.87 (0.21–3.39) 0.840	
50–59	2.19 (0.58–8.36) 0.248	
60–69	1.39 (0.37–5.20) 0.620	
70+	1.21 (0.32–4.61) 0.774	
**Sex**		
Male	Ref	Ref
Female	2.13 (1.34–3.37) 0.001	1.91 (1.06–3.45) 0.031^*^
**Level of education**		
No formal education	Ref	Ref
Primary	1.25 (0.59–2.67) 0.561	1.87 (0.77–4.56) 0.170
JSS/JHS/middle school	3.19 (1.79–5.67) <0.0001	3.08 (1.57–6.02) 0.001
SSS/SHS/O and A “level”	1.90 (0.96–3.77) 0.065	2.35 (1.04–5.33) 0.041
Technical/vocational	6.88 (2.79–16.97) <0.0001	4.62 (1.73–12.35) 0.002
Tertiary	3.07 (1.02–9.23) 0.045	5.41 (1.28–22.82) 0.020
**Occupation**		
Unemployed	Ref	
Formal sector worker	2.13 (0.47–9.50) 0.324	
Informal sector worker	1.17 (0.37–3.67) 0.789	
Retired	2.12 (0.62–7.28) 0.234	
**Marital status**		
Single	Ref	
Currently married	0.92 (0.18–4.84) 0.925	
Currently not married	0.64 (0.12–3.48) 0.603	
**Religion**		
Christianity	Ref	
Islam	0.53 (0.33–0.85) 0.009	
**Health insurance**		
Yes	Ref	
No	2.37 (0.12–49.65) 0.579	
**Type of insurance**		
NHIS	Ref	
Private insurance	0.39 (0.12–1.23) 0.109	
**Patients' satisfaction**		
Not satisfied	Ref	
Partially satisfied	0.07 (0.01–2.33) 0.135	
Satisfied	0.43 (0.03–9.11) 0.591	
	**COR**	**AOR**
	**COR**	**AOR**
**Variable**	**OR (95% CI)** ***p*****-value**	**OR (95% CI)** ***p*****-value**
**Patients' adherence to management of diabetes**		
Poor adherence	Ref	Ref
Good adherence	1.92 (1.05–3.48) 0.033	2.35 (1.11–4.96) 0.025^*^
**Flexible continuity of care**		
Low	Ref	Ref
High	7.57 (4.73–12.10) <0.001	4.62 (2.78–7.68) <0.001^*^
**Team continuity of care**		
Low	Ref	Ref
High	5.35 (3.12–9.16) 0.006	17.95 (0.94–34.22) 0.055
**Longitudinal continuity of care**		
Low	Ref	Ref
High	0.18 (0.11–0.30) <0.001	0.35 (0.19–0.64) 0.001^*^
High		0.29 (0.16–0.52) <0.001^*^

* Statistically significant.

## Discussion

This study sought to determine the extent of continuity of care between diabetic patients and their care providers as well as factors associated with relational continuity of care. The study found high continuity for all dimensions except longitudinal continuity. In addition, team continuity, flexible continuity, higher educational level and being female were associated with relational continuity of care.

It is worth noting that, a substantially high number of diabetics have their care well-coordinated among various teams of health professionals as well as experience a strong sense of harmonization among their health providers with regard to their diabetes management. This is very needful in current times of multiple conditions. Coordination of shared care among different health experts could result in positive health outcomes such as good diabetic control and reduced risk of admission suicide (24). Team continuity of care performs one of the core functions of quality healthcare and is known to be an integral part of the continuity of care (24). High team continuity experience by diabetics is an indication of high quality and satisfaction of care among patients whereas for doctors it creates a platform to increase knowledge, confidence, and skills (25). Our findings showed high experience of team continuity of care. This is a desirable attribute of continuity of care because of the many benefits of having a well-coordinated health care among health professionals. However, healthcare providers must make it a conscious effort to establish coordination by accepting the responsibility for effective communication. Furthermore, team continuity of care if not managed well can lead to undesirable outcomes. For instance, inter team conflict or conflict between a team member and a patient may break the confidence of the patient and this may lead to negative health outcomes. Similarly, diabetics having multiple health professionals attending to their health needs may sometimes be confused due to conflicting prescriptions by their care givers or other inconsistent service rendered by team members. Other factors may be inefficient sharing of patients' health history, duplication, and poor reconciliation of patients' information. These may lead to less quality of care (24, 25).

Notably, both relational and flexible continuity recorded 68.1 and 65.3% high continuity of care, respectively. This means that over 65% of diabetic patients get appropriate and consistent health support anytime it is required and more also have a high sense of affiliation with their health providers. Diabetics in this study had an appreciable number of experiencing high relational continuity. This outcome is highly appreciable since findings from other researchers project relational continuity as the most valued in primary healthcare and mental health (26). This finding connotes a well-established strong relationship between diabetics and with care providers. This level of patient–doctor friendship contributes to trust, decreases the cost of healthcare as well as increases satisfaction with health service leading to a high experience of quality care (6, 23). In addition, a study conducted in Israel reveals that patients with high relational continuity of care are more likely to achieve clinical targets. It is important to note that same study shows patients with high relational continuity had lower odds of mortality and low admission rates (23, 27). In addition, attributes of relational continuity of care such as trust, good rapport, effective communication, and confidence are known to boost the adherence to the medication of diabetics resulting in good health outcomes (24). Although most patients prefer an existing and strong affiliation with their doctors, relational continuity was not the highest in terms of all the domains of continuity. This finding agrees with a study conducted by some renowned researchers (28). It is worth to note this study shows a considerable number of diabetics in Ghana being offered services that are flexible and adjusted to their personal needs overtime. Our findings reflect the fact that most diabetes specialists adapt to care protocols to provide suitable care to the changing needs of their patients, resulting in the satisfaction of care received as well as other better health outcomes (24, 25).

Although longitudinal continuity (LCOC) of care recorded the lowest score in this study, it is consistent with other studies that have recorded LCOC score to be the lowest of all the dimensions of continuity of care (21, 29). Similarly, a study conducted in the US by Baker et al. (30) recorded a mean score of 0.61 for LCOC which is close to the average LCOC mean score of 0.5 derived from this study. This reflects the fact that only few diabetics experience an ongoing health pattern of care with the same physician at the same facility over time (31). This could also mean that high proportions of diabetic patients are seeing multiple physicians, making healthcare fragmented. This is typically experienced in Ghana since most health facilities in Ghana do group practice which makes it difficult for a patient to see the same physician continuously. Low experience of longitudinal continuity may affect the quality of care received leading to negative health outcomes. Patients who visits other physicians other than their usual physician are likely to experience the duplication of medical tests resulting to high medical cost as well as other negative consequences that stem from consulting different specialists (20). In the same sense, low experience of longitudinal continuity of care could lead to high costs for the prescription. This is proven by a study conducted in the US which demonstrated that people who experienced high longitudinal continuity for a period of 10 years had lower prescription costs as compared to those with low LCOC (17).

Most patients with low experience of LCOC from this study complained they do not get reminders for appointments with their usual providers and hence making them forget the appointment schedule. This makes them miss out on some scheduled meetings with usual health providers. In view of this, the record units of facilities should take up the responsibility of coordinating diabetes care such that regular reminders are sent to patients on their appointment days.

### Factors associated with relational continuity of care

Considerable studies have proven relational continuity of care being critical for positive recovery outcomes. Similarly, this type of continuity of care birth trust between patients and their care givers. This strong bond boost compliance with patients' medication leading to positive health outcomes (23). It is evident from the result that female subjects have higher odds of experiencing relational continuity of care than men. Many studies have shown women having higher odds of visiting health facilities than men. This trend is fueled by many natural variables such as pregnancy and its related issues, childbirth, and other gynecological and obstetrics problems thus to mention few (32). In addition, a study done in Uganda showed that healthcare was sought more by diabetic women as compared to men (33). These may possibly account for the trend observed in our studies. In addition, our results demonstrate that participant who attained the tertiary level of education is five times more likely to experience relational continuity of care as compared to those with no formal education. This may be due to the fact that people with higher educational levels are more likely to visit health facilities more and can communicate better with their care givers leading to a stronger bond (34, 35). Good communication is a good indicator of a strong relationship (23). Furthermore, people with no formal education may not have smooth communication with their clinicians due to the language barrier and other cofounders. Furthermore, our findings reveal that diabetics with higher flexible and team continuity of care are four times and 17 times more likely to experience a good relationship with their care givers. Patients who are empowered to access their doctors anytime anywhere as well as well-coordinated care given will surely have a strong existing and ongoing therapeutic relationship with their doctors (23, 25). Accounting for all other factors, it is evident from this study that high relational continuity of care of diabetics did not translate into high longitudinal continuity of care. This possibly could be that patients who have experienced low longitudinal continuity of care are more likely to experience high relational continuity of care as compared to those with high longitudinal continuity. Relational continuity is vital in ensuring that, patients develop an interpersonal relationship with their clinician on regular basis. This happens when physician–patient relationship transcends the usual contact exchange to the physician being made aware of the patient's familial circumstances. This is important in managing complications and at the same time ensuring the wellbeing of patients with diabetes. Indeed, relational continuity is beneficial in reducing hospitalization duration and reducing the use of emergency departments and also improves patient outcomes (23). This study identified a strong relationship between diabetes adherence and relational continuity among patients with diabetes. There are several possible reasons contributing to why longitudinal continuity of care has decreased the odds of relational continuity of care. Multiple referrals during health service delivery may also be a contributing factor. A well-established interpersonal relationship can make a physician in charge of a patient refer the patient when necessary since the doctor knows the familial circumstances of the patient. In this case, fragmented care received by the patient may be due to the strong interpersonal bond that exists between a patient and the physician. This could account for the low longitudinal continuity of patients with high relational continuity. Another contributing factor is the group practicing the culture of doctors. The emerging numbers of part time and salaried doctors in general practice results in seasonal rotation of practicing physicians which distorts the order of repeated visits to a particular physician over time. This finding is similar to a study conducted by Aboulghate et al. (28). Hitherto, this finding reflects the fact that most diabetics in Ghana are most likely to experience low longitudinal continuity of care.

### Study limitations

The following are some limitations of this study that should be considered when interpreting the results:

- First, the study used a cross-sectional design, which means that causal relationships cannot be established. For example, while the study found that good adherence to diabetes management was associated with high relational continuity of care, it is possible that patients who experienced high relational continuity of care were more likely to adhere to management in the first place.
- Second, the study was conducted in only three diabetic clinics in Accra, Ghana, which may limit the generalizability of the findings to other settings.
- Third, the study relied on self-reported measures of continuity of care, which may be subject to recall bias and social desirability bias.
- Fourth, the study did not assess the quality of diabetes care received by participants, which may have an impact on their perception of continuity of care.
- Finally, the study did not assess the perspectives of healthcare providers, which could provide additional insight into the factors that contribute to the continuity of care.

## Conclusion

The majority of diabetics in this study experienced high continuity of care in three of the domains of continuity. Most diabetics had their care well-coordinated among multiple health providers, were more likely to experience responsive and timely care from their health providers, and had strong interpersonal bond with their health providers. This means most health providers in the diabetic clinics used for this study have flexible plans in the management of the changing needs of patients and are able to adapt to care protocol to suit peculiar cases of patients when it faces up. Notably, patients with a high team and flexible continuity of care are 17 and four times more likely to experience relational continuity of care. Furthermore, female subjects in this study have higher odds of experiencing a continuity of care as compared to male subjects. However, the majority of diabetics in Accra experienced low longitudinal continuity of care. Considering the importance of CoC in improving quality care, there is a need for policy action on the adoption, of multidisciplinary team-based care, optimization of information technology (electronic records/data and electronic information management systems), and advocacy for family medicine. Overall, while this study provides important insights into the extent of continuity of care experienced by diabetic patients in Accra, Ghana, further research is needed to confirm these findings and identify strategies to improve longitudinal continuity of care.

## Data availability statement

The raw data supporting the conclusions of this article will be made available by the authors, without undue reservation.

## Ethics statement

The studies involving human participants were reviewed and approved by Ghana Health Service Ethics Review Committee. The patients/participants provided their written informed consent to participate in this study.

## Author contributions

VA and SKKD conceived the idea and made intellectual contributions to the conception and design. VA analyzed the data and drafted the manuscript, and it was reviewed by SKKD. Both authors read and approved the final manuscript.
